# A comparative study of the prevalence and correlates of psychiatric disorders in *Almajiris* and public primary school pupils in Zaria, Northwest Nigeria

**DOI:** 10.1186/s13034-017-0166-3

**Published:** 2017-06-08

**Authors:** Aishatu Abubakar-Abdullateef, Babatunde Adedokun, Olayinka Omigbodun

**Affiliations:** 1 Department of Psychiatry, Ahmadu Bello University Teaching Hospital Zaria, Zaria, Nigeria; 2 Centre for Child and Adolescent Mental Health, University of Ibadan, Ibadan, Nigeria; 3 Department of Epidemiology and Medical Statistics, College of Medicine, University of Ibadan, Ibadan, Nigeria; 4 Department of Psychiatry, College of Medicine, University of Ibadan, Ibadan, Nigeria

**Keywords:** Almajiris, Street children, Mental health, Zaria, Northern Nigeria

## Abstract

**Background:**

‘Almajiris’ are children and adolescents sent far away from their homes to study in Islamic schools under the care of Muslim scholars. Over the years, there has been a decline in the capacity of the scholars to cater to these pupils. Consequently, Almajiris spend significant periods of time on the streets begging and carrying out menial jobs to earn a living thereby increasing their risk for physical and mental disorders. The aim of this study was to compare the prevalence of psychiatric disorders among Almajiris and public primary school pupils in Zaria.

**Methods:**

A comparative cross-sectional design was utilized to compare 213 Almajiris and 200 public primary school children and adolescents aged between 5 and 19 years. All participants were administered a Socio-demographic questionnaire and the Schedule for Affective Disorders and Schizophrenia for School-aged Children Present and Lifetime Version (K-SADS-PL). Data were analyzed using Chi square tests and logistic regression.

**Results:**

The current prevalence of psychiatric disorders among Almajiris and public school pupils was 57.7 and 37.0% respectively. After adjusting for age and family characteristics, Almajiris were significantly more likely to have any psychiatric diagnosis, depression, enuresis, substance use, and post traumatic stress disorder but less likely to have separation anxiety disorder than the public school pupils.

**Conclusion:**

Psychiatric disorders are more prevalent among Almajiris and public primary school pupils in Northwest Nigeria than found in other prevalence studies with a significantly higher rate among the Almajiris. Joint efforts need to be made by the Government and Civil Society organizations including religious groups towards reforming the Almajiri education system and the provision of programmes aimed at reducing the prevalence of psychiatric disorders in both Almajiris and the school pupils.

## Background

Street children constitute an important social and public health challenge in both developed and developing countries of the World [53]. They are a marginalized and vulnerable group [55] and have been described by the United Nations Children’s Fund (UNICEF) as “Excluded and Invisible”. This concept is in reference to their inability to access vital resources such as health care, food and education coupled with their omission from vital statistics such as birth and death registration. This is despite their vulnerability to numerous physical and psychological hazards [53]. Ironically, street children are physically visible, living and working on roads and in public areas. Some of the health problems faced by street children include physical and sexual abuse, sexually transmitted infections, and psychoactive substance use [22].

Street children in Nigeria, show cultural and geographic diversity. In the Southern parts of Nigeria, they are typically found as ‘*street urchins*’ or ‘*area boys’* in motor parks (stations where passengers board or disembark from buses and taxis in their transit from one place to another), hawking wares or food items, or engaged in menial jobs to supplement family incomes or fend for themselves [4, 21, 34, 52]. Some features of these children include disrupted family backgrounds and poorly educated parents with large families [4]. In addition, for those street children in school, academic performance is usually poor and they are often unable to complete school due to a number of factors including school truancy, alcohol and drug abuse, having to earn some income for themselves or their family and suspension from school due to one misconduct or the other [41]. Sexual abuse [28], risky sexual behavior [43], and use of psychoactive substance [37] have been reported among street children in southern Nigeria.

By contrast, in Northern Nigeria, street children are identified as “*Almajirai* (singular *Almajiri*)”, found in groups taking Quranic lessons from Mallams (Arabic word for teachers), begging for alms, wandering the streets, performing tedious and sometimes onerous jobs in exchange for food or money [21, 34, 39]. Quranic schools have been an influential aspect of the early childhood education in Northern Nigeria [17]. They are semi-formal centers of religious education in which male children (females are rarely sent out) aged as young as 3 years are sent to Mallams faraway from their parents to acquire Islamic knowledge and learn the Holy Book [50].

The word ‘*Almajiri’* has its roots in the Arabic lingua. Its origins can be traced to the Arabic word Al-Muhajirun, which means to emigrate [12]. Traditionally, the Mallams (Teachers) were responsible for the feeding and upkeep of the Almajiris under their care [12, 32]. They often had farms of various sizes, the harvest of which was usually enough to feed their families and the Almajiris under their care. Where this was not the case, they supplemented with gifts received from members of the community. These were not in shortage due to the respect and high esteem with which Mallams are held. In recent times however, rapid urbanization and a shift from agrarian culture have brought this means of sustenance to a decline mainly through the routes of poverty and scarcity of resources.

In Hausa land, the term ‘Almajiri’ has evolved over time and in current parlance could refer to one of three categories of children: Children sent from their homes and entrusted to the care of Mallams to study the Quran; those who roam on the streets for the purpose of getting alms; and children that engage in some form of labour to earn a living [2, 8, 12]. Almajiris invited to participate in this study were children who had left home to study the Quran and are currently under the care of a Mallam.

The social profile of Almajiris includes many factors that in the long term predispose them to mental health problems. For example, they find themselves in peculiar circumstances, lacking the protection of secure family relationships having been separated from their parents as early as 3 years of age [1]. Furthermore these children usually go hungry, engage in hazardous and odd jobs in exchange for food, and are exposed to the elements on the streets. This in combination with poor physical health, lack of supervision while roaming the streets begging, and conditions of overcrowding at the Tsangayas (Quranic schools) places them at an increased risk of abuse, conditions such as anxiety, depression, post traumatic stress disorder and behavioral problems [13].

Notably however, the health of the Almajiris has received relatively little attention. The majority of existing studies and reports have focused on the educational reform of the Almajiri school system. Perhaps the only recent study that examined an aspect of the mental health of this group is that by Abdulmalik et al. which found a prevalence of psychoactive substance use of 66% [1]. In comparison to the situation in developing countries, the mental health of street youths has been the focus of several studies in the developed world. These studies have reported proportions above 80% for psychiatric disorders among homeless youth [26, 45]. Comparative studies such as those by Slesnick et al. and Kamieniecki showed twice the lifetime prevalence of psychiatric illness among homeless youth when compared with comparable controls in homes [30, 47]. Additionally, depression [36, 56] and disruptive behavior disorders [35] were significantly commoner among the homeless.

The few studies on psychopathology of homeless or street children in developing countries include a Turkish study [48] that found 61% of street children had at least one psychiatric disorder while a Ghanaian study reported that as high as 87% of homeless youth showed moderate to severe psychosocial symptoms [9]. In their study which assessed 112 Burundian male children, Crombach et al. provide evidence that psychopathology among children who had spent parts of their lives on the streets was associated with exposure to violence [19]. A recent review [22] identified physical abuse, sexual abuse, parental conflict, parental psychiatric disorder, substance use, family support and neighborhood disorganization as risk factors for psychiatric disorders. Oppong Asante found that youth’s resilience, stigma, violent behavior and suicidal ideation were associated with emotional problems among homeless youth [9].

The spectrum of mental health problems among this special class of street children, the Almajiris, is the focus of this study. The objective of this study is to determine the prevalence of psychiatric disorders among Almajiris and compare this with children in formal schools.

## Methods

### Location and participants

The study was done in Zaria, a major city in Kaduna state, North-west Nigeria. It is one of the oldest towns in Northern Nigeria. According to the 2006 National Census the population of Zaria stands at 406, 990 [38]. Statistics available in 2008 for Kaduna State show a Net Basic Education enrollment ratio of 54%, which is 9 points below the national average. It is higher for primary schools but a remarkable decline becomes apparent as the level of education increases, such as at senior secondary school level, where enrollment is 24%. In a census of all schools in Kaduna state, there were 5108 Quranic schools in the state with Zaria Local Government Area (LGA) accounting for 547 of these schools. Total enrolment in the Quranic schools in Zaria is 33,763 (Kaduna State Ministry of Education. Nigeria: Education Sector Analysis [29].

Zaria, fondly known as Zazzau was founded by a legendary warrior known as Queen Amina around the fifteenth to sixteenth century. The city is accessible by rail owing to its location on a major North–South railroad, by road through the federal highway and by air through the facilities at the Nigerian College of Aviation Technology (NCAT) located in the city centre. The main occupation of the Zaria populace is agriculture, but they engage in other activities such as embroidering ceremonial dresses. It is one of the nation’s leading producers of cotton for export and is the main ginning centre for the cotton grown in the northern Nigerian region. Zaria has a long entrenched reputation for being a centre of Islamic knowledge, making it befitting for this study. Since the nineteenth century, it has attracted pupils from all over the North of Nigeria and neighboring countries such as Niger, Mali, Cameroon and Chad for the purpose of learning and memorizing the Quran.

This was a comparative cross-sectional study evaluating mental health problems among Almajiris and public primary school pupils. Ethical approval was granted by the Health Research Ethics Committee (HREC) of the Kaduna State Ministry of Health before onset of the study. On account of the remote and sometimes scattered backgrounds of the Almajiris, obtaining parental consent would have been practically impossible. Due to these difficulties, a waiver of parental consent was sought for and granted by the committee granting ethical approval based on the principle of “no greater than minimal risk” to the participants. Permission was obtained from the Kaduna State Bureau for Religious Affairs (Islamic Matters) and the Kaduna State Ministry of Education to interview Almajiris and public primary school pupils respectively. For the purpose of this study, the guardian of an Almajiri was considered to be the Mallam under whose care he was. All Almajiris aged 5–19 years in the selected Quranic schools who assented and whose guardian gave informed consent for them to participate in the study were included. The comparison group comprised an equal number of assenting pupils selected from nearby public primary schools whose parents or guardian gave informed consent for their inclusion in the study. Participants signed or thumb printed an assent form if they were less than 18 years and a consent form if they were 18 years or older. Consent forms were made available to parents and guardians of public school pupils to peruse two days to commencement of the study. Signature or thumbprint appended to these forms indicated consent for the child or ward to be interviewed.

One public school pupil who had gross features of intellectual deficiency was excluded from the study due to inability to understand the questions. He was replaced with another pupil selected at random from the sample frame. All other participants were proficient in either English or Hausa.

### Sample size and sampling procedures

The minimum number of children studied was determined assuming a 5% chance of Type 1 error, 80% power and 10% non-response rate. Assuming a 15% difference between the two populations and 66% as an estimate of psychoactive substance use from a previous study [1], 182 children was determined to be minimum sample size. 213 Almajiris and 200 children in public schools were eventually studied.

A multistage sampling method was employed for selection of participants in the two groups. For the Almajiris, Zaria was divided into wards and from this sample frame 3 wards were randomly selected for the study in the first stage. In the second stage, all Almajiri schools in the selected wards were identified and one was selected from each through ballot. Though the proposed minimum sample size was 182 per group, there were 213 Almajiris between the ages of 5 and 19 in the three selected schools (85, 58 and 70 Almajiris respectively) and all of them were interviewed. For Almajiri pupils whose age could not be readily identified, the Mallams and older pupils assisted the interviewers in age estimation by asking simple questions such as how old they were when they left home and how many years they had spent at Almajiri school. This was done in a bid to ensure they fell within the age range for inclusion into the study. Mallams in the Quranic schools gave permission for their pupils to be interviewed only during the weekends (Saturdays and Sundays) so as to reduce disruption to their off-school days which are usually observed on Thursdays and Fridays.

Concerning selection of children in the public schools, a list of schools in the selected wards was obtained from the Zaria Local Government Education Authority, three of which were then selected: Adamu Dikko, Bashir Adamu and Tsoho Abdullahi Local Education Authority (LEA) Schools. The comparison group was selected such that each LEA school was closest to the Almajiri School studied. Seventy-two male pupils were selected for participation from each school, as all the three schools selected had roughly the same number of children enrolled. Class registers containing the names and gender of pupils in Primaries 1–6 were obtained and 12 male pupils randomly selected per level thus reaching the required total of 72 pupils. Interviews in the primary schools were carried out on three separate working days spanning 2 weeks. Public primary schools were chosen as a comparison to factor in as closely as possible, the same socio-economic circumstances.

## Materials

Two main instruments were used for data collection: a socio-demographic characteristics questionnaire adapted from the Global School-based Health Survey (GSHS) Nigeria Questionnaire and the Schedule for Affective Disorders and Schizophrenia for School aged Children-Present and Lifetime Version (K-SADS-PL). The Socio-Demographic Questionnaire was employed to gather relevant information about the participants and their family characteristics. It had three sections with the first section obtaining information about age, religion, nationality and state of origin. The second section obtained information about the family background of the participants including family type (monogamous or polygamous), parents’ marital status, family size and parents’ level of education while the third section was specific to the Almajiris and included questions on age at first leaving home for Quranic school, their source of income, meals and where they slept. Variables assessing if participants had sustained injury, being in a physical fight or being bullied (all in the 12 months preceding study) were included in this section and had been adapted from the GSHS-Nigeria.

The K-SADS-PL is a semi-structured diagnostic interview for children [31]. It is designed to assess current and past episodes of psychopathology in children and young persons according to the third and fourth edition of the Diagnostic and Statistical Manual of Mental Disorders (DSM-III-R and DSM-IV) criteria. Though the K-SADS-PL interview includes additional information obtained from the parents, for the purpose of this study, it was administered on the participants only. This was in view of the difficulties that would have been encountered with attempting to trace parents of the participants especially the Almajiris. The K-SADS-PL is divided into two parts, the Screen Interview and Diagnostic Supplements. The Screen Interview evaluates for primary symptoms of the different diagnosis groups. Symptoms in the screen Interview are rated for current and most severe past. Symptoms are rated negative for current and past episode if the child has never experienced them. Affirmative answers are further probed as to when those symptoms were present and rated accordingly. The Diagnostic Supplement has a list of probes and criteria to assess for current or lifetime history of psychiatric disorders. The K-SADS-PL assesses exposure to traumatic events as part of the screen for Post-Traumatic Stress Disorder. The instrument, upon completion of its administration yields a definitive psychiatric diagnosis. Participants who had significant symptoms on application of the screen interview were then taken through the corresponding diagnostic supplements for confirmation. The K-SADS-PL probes were extracted into a separate document for ease of translation and administration. All instruments used in the study were translated to Hausa using the back-translation method by a psychiatrist and linguist with proficiency in English and Hausa. The instruments were first translated into Hausa by a Hausa linguist and subsequently, an independent psychiatrist who was blind to the original instrument translated this Hausa version back to its original language, English. The original and the newly translated English versions were then compared by the authors. Any inaccuracies and mistranslations were made known to the Hausa linguist who made a fresh translation of the problematic question to Hausa and the independent psychiatrist translated this to English. This process was repeated until the newly translated English version came as close as possible to the original document.

Four research assistants were trained on the administration of the socio-demographic questionnaire while the author, AA administered the K-SADS-PL which is designed to be used by trained clinicians. Each item and its alternative were read out to the child in Hausa or English depending on his preferences, options chosen by the participant were then marked on the instrument by the author or research assistant. Almajiris were interviewed in their Quranic schools. The *Zaure*, (a large ante-chamber in traditional Hausa architecture) was made available to the interviewers for use in these schools. In the first two public primary schools visited, empty classrooms were cleaned and set-up for the interviews, while in the third school, the interviews were conducted in the school library. One pupil was interviewed at a time, in an area away from the others, to ensure a maximum level of privacy and confidentiality. There were no names or identification markers on the questionnaires so as to ensure anonymity.

### Data analysis

Data collected were analyzed using the Statistical Package for Social Sciences (SPSS) software version 21 (SPSS-21). Comparison of categorical variables such as respondents’ socio-demographic characteristics and psychiatric diagnosis between the two groups were tested using Chi square and Fisher’s exact tests. The mean number of traumatic events was compared between the groups using the independent samples t test. Univariate logistic regression analyses were carried out to estimate odds ratios comparing psychiatric diagnoses between Almajiris and children in public schools. Adjustments for age, family type, marital status of parents, father and mother’s education, and number of parents’ children were made in multivariable logistic regression analyses for selected psychiatric diagnoses (based on an appreciable number of children that had those conditions) including study group (Almajiri versus public school children) as the main independent variable. Crude and adjusted odds ratios (ORs) and their 95% confidence intervals were reported for univariate and multivariable logistic regressions respectively. Hosmer–Lemeshow goodness of fit tests was used to assess model fit. Level of significance was at 5%.

## Results

### Demographic information of participants

A total of 213 Almajiris and 200 public school pupils were involved in the study. The mean age of the Almajiris in years was significantly higher than that of public school pupils (13.1 ± 3.5 vs. 10.9 ± 2.9, t = −6.69, df = 411, p < 0.001, 95% CI −2.90 to −1.58). All the participants indicated that their family religion was Islam. About a quarter (25.8%) of participants in the Almajiri group indicated they were non-Nigerian, compared to 3% of the public school pupils. All the non-Nigerians were from Niger Republic which borders Nigeria to its North. All other participants indicated they were from Northern parts of Nigeria although a significantly higher proportion of Almajiris than public school pupils (19.5% vs. 4.2%) hailed from the North-Central region which is outside the region where the study was conducted.

Table 1 shows the distribution of selected characteristics of Almajiris and the public school pupils.

**Table 1:** Comparison of selected socio-demographic characteristics of Almajiris and the public school pupils

Variable	Almajiris N = 213 (%)	Public school pupils N = 200 (%)	χ^2^	p value
Age in 5 year categories				
5–9	29 (13.6)	68 (34.0)	74.48	<*0.001*
10–14	86 (40.4)	112 (56.0)		
15–19	63 (29.6)	19 (9.5)		
Don’t know	35 (16.4)	1 (0.5)		
Family type				
Monogamous	61 (28.8)	82 (41.2)	6.99	*0.008*
Polygamous	151 (71.2)	117 (58.8)		
Marital status of parents				
Living together	189 (89.6)	172 (86.4)	1.06	0.589
Divorced/separated	8 (3.8)	11 (5.5)		
Parent(s) deceased	14 (6.6)	16 (8.0)		
Father’s children				
1–4	28 (13.1)	22 (11.1)	0.50	0.779
5–9	106 (49.8)	104 (52.3)		
10+	79 (37.1)	73 (36.7)		
Mother’s children				
1–4	57 (26.8)	35 (17.6)	5.12	0.077
5–9	126 (59.2)	135 (67.8)		
10+	30 (14.1)	29 (14.6)		
Father’s level of education				
No formal education	32 (15.1)	10 (5.1)	101	<*0.001*
Quranic school	147 (69.3)	62 (31.3)		
Primary school	16 (7.5)	47 (23.7)		
Secondary and higher	17 (8.0)	79 (39.9)		
Mother’s level of education				
None	44 (20.8)	24 (12.1)	88.63	<*0.001*
Quranic school	153 (72.2)	79 (39.7)		
Primary	9 (4.2)	46 (23.1)		
Secondary and higher	6 (2.8)	50 (23.1)		

Figures in italics indicate significant values

Almajiri pupils had a significantly higher proportion coming from polygamous homes, and had fathers and mothers with lower education than public school participants. The two groups were not significantly different concerning marital status of parents, number of mother’s or father’s children.

### Traumatic events among participants

Table 2 shows that more Almajiris reported having ever been involved in a car accident, ever witnessed an accident and ever been physically abused. The mean number of traumatic events was significantly higher among Almajiris (1.38, SD = 1.05) compared to public school pupils (mean = 0.87, SD = 0.83) (p < 0.001).

**Table 2:** Exposure to Traumatic Events among Participants (N = 413)

Traumatic event	Almajiris	Public school pupils	χ^2^	p value
	N = 213 frequency (%)	N = 200 frequency (%)		
Vehicle accident^a^	34 (16.0)	3 (1.5)	26.15	<*0.001*
Witness accident	38 (17.8)	2 (1.0)	33.09	<*0.001*
Witness of disaster	43 (20.2)	7 (3.5)	26.63	<*0.001*
Witness of crime	0 (0.0)	2 (1.0)		0.231*
Victim of violence	1 (0.5)	1 (0.5)		1.000*
Traumatic news	158 (74.2)	151 (75.9)	0.159	0.690
Witness to domestic violence	0 (0.0)	2 (1.0)		0.231*
Physical abuse	23 (10.8)	3 (1.5)	14.92	<*0.001*
Sexual abuse	0 (0.0)	2 (1.0)		0.231*

Figures in italics indicate significant values

* Indicates Fisher’s exact statistic

^a^Including motorcycle and bicycle accidents

### Prevalence and pattern of psychiatric diagnosis

The differences in psychiatric diagnosis on K-SADS-PL between the two groups of children are shown in Table 3. A significantly higher proportion of Almajiris (57.7%) had an identifiable diagnosis on the K-SADS-PL compared to their public school counterparts (37.0%, p < 0.001). Concerning specific conditions, a higher proportion of Almajiris compared to their public school counterparts met the criteria for a diagnosis of depression, generalized anxiety disorder, enuresis, substance use and post traumatic stress disorder. However public school pupils were significantly more likely than Almajiris to meet the diagnosis for Separation Anxiety and Obsessive Compulsive Disorders. No significant differences were noted in the prevalence of mania, psychosis, social phobia, panic attacks, agoraphobia, encopresis, attention deficit hyperactivity disorder (ADHD), oppositional defiant disorder, and conduct disorder. None of the participants met the criteria for Anorexia Nervosa or Bulimia Nervosa. Logistic regression analysis was done for those diagnoses with a sizeable number of participants with the condition in both groups.

**Table 3:** Bivariate and multivariable comparisons of psychiatric diagnoses on K-SADS-PL between Almajiris and public school pupils

K-SADS-PL diagnosis	Crosstabs		Univariate logistic regression	Multiple logistic regression
	% with diagnosis	p**	Crude OR (95% CI)	Adjusted OR*** (95% CI)
Any condition				
Almajiris	123 (57.7)	<*0.001*	*2.33 (1.57*–*3.46)*	*3.11 (1.79*–*5.41)*
Public school pupils	73 (37)			
Depression				
Almajiris	49 (23.0)	<*0.001*	*3.40 (1.86*–*6.21)*	*2.93 (1.27*–*6.76)*
Public school pupils	16 (8.1)			
Mania				
Almajiris	1 (0.5)	0.611*		
Public school pupils	2 (1.0)			
Psychosis				
Almajiris	5 (1.2)	0.062*		
Public school pupils	0 (0.0)			
Panic attacks				
Almajiris	1 (0.5)	0.201*		
Public school pupils	4 (2.0)			
Separation anxiety				
Almajiris	3 (1.4)	<*0.001*	*0.09 (0.03*–*0.29)*	*0.14 (0.03*–*0.64)*
Public school pupils	28 (14.0)			
Social phobia				
Almajiris	2 (0.9)	0.269*		
Public school pupils	5 (2.5)			
Agoraphobia				
Almajiris	1 (0.5)	0.059*		
Public school pupils	6 (3.0)			
GAD				
Almajiris	34 (16.0)	*0.015*	*2.16 (1.15*–*4.05)*	*1.92 (0.79*–*4.70)*
Public school pupils	16 (8.1)			
OCD				
Almajiris	0 (0.0)	0.025*		
Public school pupils	5 (2.5)			
Enuresis				
Almajiris	43 (20.2)	*0.012*	*2.02 (1.16*–*3.53)*	*3.42 (1.59*–*7.36)*
Public school pupils	22 (11.1)			
Encopresis				
Almajiris	0 (0.0)	0.482*		
Public school pupils	1 (0.5)			
ADHD				
Almajiris	1 (0.5)	0.059*		
Public school pupils	6 (3.0)			
ODD				
Almajiris	3 (1.5)	1.000*		
Public school pupils	3 (1.4)			
Conduct disorder				
Almajiris	1 (0.5)	1.000*		
Public school pupils	1 (0.5)			
Tic disorders				
Almajiris	0 (0.0)	0.482*		
Public school pupils	1 (0.5)			
Substance use				
Almajiris	12 (5.6)	*0.010*	*5.85 (1.29*–*26.48)*	*10.05 (1.20*–*84.06)*
Public school pupils	2 (1.0)			
PTSD				
Almajiris	21 (9.9)	*0.021*	*2.60 (1.12*–*6.01)*	*6.20 (1.90*–*20.19)*
Public school pupils	8 (4.0)			

Figures in italics indicate significant values

* Based on Fisher’s Exact tests

** Based on Chi square tests; 213 Almajiris and 198 public school pupils included in the cross-tabulations

*** Adjusted for age, family type, education of father, educational level of mother, number of mother’s children, number of father’s children, number of mother’

As shown in Table 3, the adjusted odds of the diagnosis of depression, enuresis, substance use and PTSD remained significantly higher among Almajiris while separation anxiety was significantly more likely among public school children. The adjusted odds ratio for Generalized Anxiety Disorder was however not significant.

### Correlates of psychiatric diagnosis

Variables found to be significantly associated with an Almajiri having a psychiatric diagnosis on the K-SADS-PL on bivariate analysis are shown in Table 4. These were mother’s highest level of education, having to go hungry, sustaining serious injury, involvement in a physical fight, being bullied in the last 30 days and visiting home less than three times a year.

**Table 4:** Correlates of Psychiatric Diagnosis among Almajiris (N = 213)

Variable	Psychiatric diagnosis on K-SADS-PL		χ^2^	p value
	Yes frequency %	No frequency %		
Mother’s education				
None	32 (72.7)	12 (27.3)	6.289	*0.043*
Quranic	85 (55.6)	68 (44.4)		
Some formal education	6 (40.0)	9 (60.0)		
Often goes hungry				
Yes	84 (64.1)	47 (35.9)	5.244	*0.022*
No	39 (48.1)	42 (51.9)		
Sustained injury				
Yes	69 (67.0)	34 (33.0)	7.312	*0.007*
No	53 (48.6)	56 (51.4)		
Involved in a physical fight				
Yes	68 (65.4)	36 (34.6)	5.133	*0.023*
No	54 (50.0)	54 (50.0)		
Bullied in the last 30 days				
Yes	75 (65.8)	39 (34.2)	6.503	*0.011*
No	48 (48.5)	51 (51.5)		
Child visited parents in the last 12 months				
<3 times	103 (74.6)	35 (25.4)	47.74	<*0.001*
3 or more times	18 (25.0)	54 (75.0)		

Figures in italics indicate significant values

## Discussion

This study has shown that overall, psychiatric diagnoses were more common among Almajiris using the K-SADS-PL. Additionally, diagnoses of depression, enuresis, substance use, and PTSD were more likely and separation anxiety less likely among Almajiris compared to public school pupils. To our knowledge this is one of the very few sub-Saharan African studies studying the mental health of street children. Most noteworthy, this study has focused on a special group of street children attending Islamic schools in sub-Saharan Arica. Our study provides information about the health of this group of children, an area that has been accorded little attention.

The proportion of participants having any mental health problems among the Almajiri and public school pupils (57.7 and 37% respectively) are much higher than 11. 4% seen in a primary care unit in Ilorin, North central Nigeria [51] and 20% in a similar clinic in South West Nigeria [25]. This could be attributed to difference in study populations. However, studies of street children in developing countries such as in Ghana [9] and Turkey [48] have reported similar high rates of psychiatric morbidity of 87 and 61% respectively.

Almajiris had significantly higher odds of depression, enuresis, substance use and post traumatic stress disorder than public school pupils after adjusting for age and some family characteristics. Almajiris typically live a life of uncertainty, they are not sure where the next meal will come from, they lack the basic necessities of life and overall their socio-economic circumstances are pervasively dire. They are also exposed to stressors such as traumatic events more often than other children. However, when we adjusted for exposure to traumatic events in the logistic regression model of depression on study group (not shown in the results), the higher odds still remained. The cross sectional nature of the data makes conclusions about the interrelationships between these variables difficult.

A higher prevalence of depression and Post Traumatic Stress Disorder has similarly been reported amongst homeless adolescents [13]. Depression has also been shown to be higher among homeless children in a US study compared to controls [36]. A previous study of major depressive disorder among male adolescents in a Nigerian secondary school reported values of 5.5% which is close to the prevalence of 8.1% found among public school pupils in our study [6].

The prevalence of substance use among Almajiris of 5.6% found in our study differs from that which Abdulmalik and colleagues found five years previously in North East Nigeria where 66.6% of Almajiris were engaged in the use of a psychoactive substance [1]. While this may be a reflection of the true situation, it may be partly explained by differences in the focus of study and methodology. The study by Abdulmalik et al. assessed Almajiris solely for psychoactive substance use using the Global School-Based Health Survey (GSHS) Questionnaire and the WHO Student Drug Use Questionnaire which has been validated for use among Nigerian students [3]. It is thus possible the instruments were more sensitive in picking up drug use than the K-SADS-PL.

The occurrence of behavioral problems including oppositional defiant disorder, conduct disorder and ADHD were strikingly low. Only one Almajiri (0.5%) met the criteria for a diagnosis of conduct disorder. This is lower than 4.2% reported among secondary school adolescents in Northwest Nigeria [11]. It is also lower than 6.1% reported by Gureje et al. and 9.3% by Adewuya in Southwest Nigeria [5, 25]. Also, previous studies have shown significantly higher occurrence of disruptive behaviours among homeless children compared to those living in homes [35, 56]. The relatively low rates of behavioural disorders may be due to the reliance of this study on self-reports rather than parent, teacher or peer evaluations. Observations have shown that children are better at disclosing their internalizing problems while externalizing behaviours are better picked up by reports from parents, teachers or peers.

Public school pupils were about eight times more likely to have separation anxiety than Almajiris. About 14% of the public school pupils in this study had separation anxiety, much higher than the 2.1% from a previous study among Nigerian in-school adolescents in Southwest Nigeria [6]. This finding could be explained by the recent tide of insurgency in the Northern parts of Nigeria. In the immediate vicinity where the study was conducted, there have been three bomb blasts in the last 2 years and about 3 other such blasts in the capital city, Kaduna which is about 80 km away from the study site [7, 49]. There have also been a number of killings and kidnappings of notable people in the same areas [20, 44, 46]. The Islamic sect ‘Boko Haram’ which operates in Northern Nigeria has recently waged war against any form of Western style education, sometimes killing male students found in formal schools [14, 24] or abducting girls in such schools [15]. Further studies are required to investigate the effects of the insurgency on school children, so that appropriate mental health interventions can be offered to children attending formal schools in the Northern part of the country. An explanation for the comparatively lower diagnosis of separation anxiety among Almajiris is that these children have been separated from their families, some from as early as 3 years of age, thus they are unlikely to have the levels of attachment requisite for the development of separation anxiety. These high rates of separation anxiety among public school pupils have ominous implications for child education in a region with low literacy levels.

There were higher odds of exposure to traumatic events, accidents and disaster among Almajiris compared to public school pupils. The reason may be due to the significant periods spent by the Almajiris on the streets begging for a livelihood, more often than not unaccompanied by any adult, and are thus more likely to be involved in or witness such events. Traumatic events have been linked with depressive symptoms in studies conducted in Nigeria and a higher likelihood of developing these symptoms was noted if the event directly affected the child [42]. This could also explain the high rates of depression found among the Almajiris. The higher reports of physical abuse experienced by Almajiris is consistent with risks associated with their source of income and meals, where some of them are engaged in paid work in some households [23]. Corporal punishment is also commonly practiced as a means of discipline in Quranic schools, and overall religious and cultural beliefs may encourage the use of force as a corrective measure in children [42].

Mothers of Almajiris had lower levels of formal education than those of public school pupils. Dating back to the time when the country had regional governments, the Western Region provided free education for its people but this was not replicated in the North. In the North, much emphasis was placed on Quranic and Arabic education and has been postulated as a reason for the perpetuation of the Almajiri system of education in its current state in previous studies [23]. There was a higher likelihood to go hungry among Almajiris. This has huge implications for their physical and mental health. Nutrients derived from food are necessary for the proliferation of cells and tissues in the body which lead to visible growth. The brain experiences rapid proliferation during childhood [33], and at the same time, fundamental cognitive and interpersonal skills are being acquired. The child’s vocabulary, problem solving skills, attention and motor coordination all increase significantly during this period. Thus there is a likelihood of malnutrition and attendant risks of poor cognitive development where nutrition is inadequate either in terms of the quantity or quality [10, 16, 18].

Approximately half of the Almajiris reported being bullied in the previous month. Almajiris are often perceived in the society with negative connotations such as ‘miscreants’, ‘dirty children’ and frequently chased away by adults. Earlier reports have highlighted how they suffer stigma and hostility from pupils of public schools during attempts at integrated education [27, 54]. About half of them had not visited their parents or guardians in the last 12 months. Leaving the protective family enclosure at their formative years, coupled with the lack of care and supervision encountered in the Quranic schools and diminished parent–child interaction is likely to exacerbate the vulnerability of these young children.

Some differences in the socio-demographic and family characteristics of the two groups studied deserve comments. The finding of older children in Almajiri schools is not unusual as the Almajiri system of education is an unstructured program with graduation dependent on a complete grasp of the Holy Quran. This means that sometimes the period of tutelage may extend for many years [40]. Notably, a modest proportion of Almajiris couldn’t tell their ages spontaneously compared to the public school pupils. For such Almajiris, their ages had to be deduced by some extrapolations such as adding the number of years they had been at Almajiri School to their age at leaving home. Though their ages were eventually deduced, for the purpose of analysis they were left to constitute the group which didn’t know their ages. This was borne out of the authors’ belief that it was a possible indication of reduced parent–child interaction among the Almajiris. It is expected that a child who lives with or maintains adequate interaction with his parents would at one time or the other be made aware of his age.

### Limitations

This study has some limitations. First is the issue of the appropriateness of the comparison group. Several characteristics such as family background are different between the two groups making comparability difficult. However we adjusted for selected family characteristics in a multiple logistic regression model. Secondly, some variables previously reported to influence the risk of psychiatric disorders such as history of mental illness and substance use were not collected due to difficulty in getting access to the parents of the Almajiris. These parents’ characteristics would have been adjusted for on the multiple regression model. Thirdly the exclusion of Almajiris that couldn’t understand the questions could influence the prevalence of psychiatric diagnoses if those excluded had characteristics that could predispose them to psychiatric disorders.

A fourth limitation is the cross-sectional nature of this study that limits the understanding of relationships between the prevalence of psychiatric morbidity and factors such as family characteristics. Longitudinal studies are needed that will enroll Almajiris from the point of departure from their families through time spent on the streets. Finally, data was collected through self-reports, as caregivers and parents were not available to provide an alternative source of information for the Almajiris, thus the reported rates for seemingly negative attributes such as behavioral problems (oppositional defiant disorder, conduct disorder, attention deficit hyperactivity disorder and substance abuse) in this population may not be a true estimate. Also, due to multiple significance testing, there is the chance of inflated type 1 errors, and larger p values should be interpreted with caution.

## Conclusion

The findings from this study indicate that the prevalence of psychiatric disorders among Almajiris was higher than in the public school population. Depression, Enuresis, Substance Use and Post Traumatic Stress Disorder were higher in Almajiris than public school pupils. The dire living circumstances of the Almajiris require earnest and resolute efforts towards improving their socio-economic status and providing them with formal education. Attempts at reforming the Almajiri schools by the Government should be supplemented with measures to promote physical and mental health, including general health education, screening, early detection and management of pupils at risk of developing psychiatric disorders. Leaving the protective family unit and diminished parent–child interaction is likely to exacerbate the vulnerability of these young children, thus the current practice where they are discouraged from visiting home should be abolished in order to help them maintain a secure and nurturing bond with their families.

## Abbreviations

K-SADS-PL: Schedule for Affective Disorders and Schizophrenia for School-aged Children-Present and Lifetime Version
UNICEF: United Nations Children’s Fund
LEA: Local Education Authority
DSM-IIIR: Diagnostic Statistical Manual Third Revision
DSM-IV: Diagnostic Statistical Manual Fourth Version
ADHD: Attention Deficit Hyperactivity Disorder
PTSD: Post Traumatic Stress Disorder
GSHS: Global School-Based Health Survey
GAD: Generalised Anxiety Disorder
OCD: Obsessive Compulsive Disorder
ODD: oppositional defiant disorder
